# Draft Genome Sequence of Cold-Tolerant Pseudomonas sp. Strain NKUCC02_KPG, Isolated from Old Alexandria Reservoir in Northern Kentucky, USA

**DOI:** 10.1128/mra.00909-22

**Published:** 2022-10-06

**Authors:** Gillian Boone, Kylie Griffey, Joshua T. Cooper

**Affiliations:** a Department of Biological Sciences, Northern Kentucky University, Highland Heights, Kentucky, USA; University of Southern California

## Abstract

We report the draft genome of cold-tolerant Pseudomonas sp. strain NKUCC02_KPG, which was isolated from a lake in Kentucky, namely, Old Alexandria Reservoir. This strain contains several genes associated with cold adaptation and is characterized by a G+C content of 58.21% and a total length of 5,187,984 bp.

## ANNOUNCEMENT

Cold-adapted species of Pseudomonas have commonly been isolated from freshwaters receiving urban stormwater runoff (1), from activated sludge (2), and from pristine cold fjord waters in the Arctic (3). They have also been identified from cold spoiled foods (4, 5) and soils (5). Several cold-adapted Pseudomonas strains have been shown to degrade antibiotics such as sulfamethoxazole (2) and to develop antimicrobial resistance (6, 7). Isolation and identification of species in this genus are important for determination of the potential potability of freshwaters, detection of antimicrobial-resistant groups, and elucidation of genomic adaptions to cold environments.

Pseudomonas sp. strain NKUCC02_KPG was isolated from a surface water grab sample obtained in January 2021 at the shoreline of Old Alexandria Reservoir (Alexandria, KY) (N 38.961166, W 84.368969) using a sterile Whirlpak bag. Strain NKUCC02_KPG was isolated from serial dilutions spread on tryptic soy agar that had been incubated at 25°C for 48 h. A single colony was grown in tryptic soy both at 25°C and genomic DNA extracted using the UltraClean microbial DNA isolation kit (Qiagen, Germantown, MD, USA) following the manufacturer’s protocol. DNA was quantified using a Qubit v3.0 fluorometer and the broad-range kit and was sequenced at the Microbial Genome Sequencing Center (MiGS) (Pittsburgh, PA, USA) using the Illumina NextSeq 2000 platform (2 × 150 bp), with libraries prepared using the Nextera DNA library preparation kit. Sequencing produced 3,411,987 paired-end reads and total of 1,010,005,316 bp, which were trimmed and filtered using fastp (8) and then assembled using Shovill v1.1.0 (9). The genome coverage was 193×, as calculated using BBMap v38.90 (10). The assembled genome was annotated using the NCBI Prokaryotic Genome Annotation Pipeline (PGAP) v5.2 (4). Default parameters were used for all software unless otherwise specified.

The draft genome is 5,187,984 bp and consists of 55 contigs, with a G+C content of 58.21% and an *N*_50_ value of 223,184 bp; the largest contig is 567,393 bp. The genome was estimated to contain 4,751 protein-coding genes, and 3 noncoding RNAs, 11 rRNAs, and 65 tRNAs were detected. CheckM (11) predicted that the genome is 99.97% complete, with 0.54% contamination, and the strain was tentatively identified as Pseudomonas psychrophile, with a match of 99.29%, using the GTDB-Tk classify workflow (12) within KBase (13). Taxonomic relations were explored among other Pseudomonas species using JSpeciesWS (14), and Tetra Correlation Search was used to select genomes sharing >95% identity. Pairwise Tetra Correlation Search analysis indicated close relationships to P. psychrophila (DSM 17535 and CF149) and Pseudomonas deceptionensis (DSM 26521 and LMG 25555), with values of 0.99532 to 0.99211. Average nucleotide identity based on BLAST+ (ANIb) and digital DNA-DNA hybridization (dDDH) calculated with formula d4 by TYGS (15) indicated that strain NKUCC02_KPG likely represents a novel cold-tolerant species within the genus Pseudomonas, with only 91.5% ANIb to Pseudomonas psychrophila DSM 17535, and the strain demonstrated only 46.8% similarity to P. psychrophila. These values are below recommended species delimitation values, i.e., ANIb values of >95 to 97% and dDDH values of >70%. Four predicted cold-shock proteins and two cold-shock domain-containing proteins (CspD, and CspD-like) were annotated. ResFinder v4.1 (16) indicated no acquired resistance to antimicrobials that might be present in the aquatic cold-tolerant Pseudomonas strain.

### Data availability.

This whole-genome shotgun project has been deposited in DDBJ/ENA/GenBank under the accession number JAHKRC000000000. The version described in this paper is version JAHKRC000000000.1. This accession number is under BioProject accession number PRJNA734631 and BioSample accession number SAMN19515367. Raw reads are available in the SRA under accession number SRR15168785. A log file output of the assembly from Shovill v1.1.0 and the graph file (.gfa) are available at FigShare (https://doi.org/10.6084/m9.figshare.14999787.v1).
